# Research summary, possible mechanisms and perspectives of gut microbiota changes causing precocious puberty

**DOI:** 10.3389/fnut.2025.1596654

**Published:** 2025-04-25

**Authors:** Maorong Bao, Rui Wu, Jingwei Li, Runan Tang, Cui Song

**Affiliations:** ^1^Chongqing Key Laboratory of Pediatrics, Ministry of Education Key Laboratory of Child Development and Disorders, National Clinical Research Center for Child Health and Disorders, Children’s Hospital of Chongqing Medical University, Chongqing, China; ^2^Ba’nan Hospital Affiliated to Chongqing Medical University, Chongqing, China

**Keywords:** precocious puberty, gut microbiota, HPG axis, dysbiosis, microbial therapeutics

## Abstract

The increasing global incidence of precocious puberty, linked to environmental, metabolic, and genetic factors, necessitates innovative therapies beyond gonadotropin-releasing hormone (GnRH) analogs. Accumulating evidence implicates gut microbiota dysbiosis as a pivotal regulator of pubertal timing via interactions with hormone metabolism (e.g., estrogen reactivation via *β*-glucuronidase), neuroendocrine pathways (nitric oxide signaling), and immune-inflammatory responses. This review delineates taxonomic alterations in central precocious puberty (CPP) and obesity-related subtypes, including *Streptococcus* enrichment and *Alistipes* depletion, alongside functional shifts in microbial metabolite production. Mechanistic insights highlight microbiota-driven modulation of the hypothalamic–pituitary-gonadal (HPG) axis, leptin/insulin dynamics, and epigenetic regulation. Emerging interventions-probiotics, fecal microbiota transplantation (FMT), and dietary modifications-demonstrate efficacy in preclinical models and early clinical studies for delaying puberty onset and restoring hormonal balance. Translational efforts to validate these strategies are critical for addressing the clinical and psychosocial challenges posed by precocious puberty, positioning gut microbiota modulation as a novel therapeutic frontier in pediatric endocrinology.

## 1 Introduction

Precocious puberty, characterized by the premature emergence of secondary sexual traits and a rapid increase in height during childhood, has seen a noticeable uptick in prevalence worldwide in recent times. This condition does not just disrupt normal physical development in children; it is also intricately linked to a host of health concerns, including metabolic disorders, cardiovascular complications, and psychological challenges. Therefore, exploring its etiology and effective interventions has become a significant topic in pediatric endocrinology.

Gonadotropin-releasing hormone (GnRH) analogs are the standard treatment for central precocious puberty (CPP). However, challenges such as prolonged treatment duration, high costs, psychological distress related to injection formulations, and local injection site reactions persist. Consequently, there remains a clinical need for new oral therapies. Recent research suggests gut bacteria significantly influence puberty initiation by regulating hormone metabolism, energy balance, and immune responses, as well as through pathways like the gut-brain axis and gut-endocrine axis.

However, the relationship between gut microbiota and precocious puberty is not yet fully understood. This review aims to summarize the potential role of gut microbiota in precocious puberty, presenting its impact through mechanisms such as metabolism, neuroendocrine pathways and immunity. Furthermore, it examines the constraints of existing research and contemplates future trajectories. The aim is to offer fresh perspectives for the accurate diagnosis and treatment of precocious puberty, and to boost the utilization of gut microbiota in the treatment of endocrine disorders.

## 2 Precocious puberty overview

In China, precocious puberty pertains to the development of breast tissue in girls prior to reaching 7.5 years old, or the occurrence of menarche before the age of 10.0 in boys, it means testicular development before they turn 9.0 (1). In recent years, the global incidence of precocious puberty has been rising, particularly in Asia. CPP, which constitutes around 90% of all cases, is 15–20 times more common in girls compared to boys (2). Notably, 90% of female CPP cases are idiopathic (3). This condition not only leads to the early appearance of secondary sexual characteristics but also advances bone age, compromised adult height, and an increased risk of metabolic syndrome. If not treated promptly, it may cause psychological issues and social dysfunction in affected children.

Long-term, precocious puberty is associated with an elevated risk of breast cancer, obesity, type 2 diabetes, cardiovascular diseases, and overall mortality (4–6). The core mechanism of CPP is the premature activation of the hypothalamic–pituitary-gonadal (HPG) axis. Under normal circumstances, the hypothalamus secretes GnRH, stimulating the anterior pituitary to release follicle-stimulating hormone (FSH) and luteinizing hormone (LH), which act on the gonads to promote the secretion of sex hormones. This process maintains normal gonadal development and reproductive system function through a negative feedback mechanism.

In CPP, early HPG axis activation triggers premature GnRH, LH, and FSH release, causing accelerated gonadal maturation. This process is influenced by neurotransmitters, metabolic signals, epigenetic modifications, as well as environmental and genetic factors (7). Risk factors for precocious puberty include obesity, genetic background, dietary habits, exposure to endocrine-disrupting chemicals, blue light exposure, anxiety and fear, vitamin D deficiency, and increased use of disposable products (8, 9). Mutations in genes such as *KISS1*/*KISS1R* and *MKRN3* et al. are associated with familial CPP (10).

## 3 Study on the association between gut microbiota and both gonadal development and precocious puberty

As research on the link between gut microbiota and precocious puberty grows, there is more clinical evidence suggesting that the composition and function of the gut microbiota may be closely related to gonadal development and the onset and progression of precocious puberty. This is mainly reflected in the following aspects:

### 3.1 Differences in gut microbiota across life stage and gender

There are over 35,000 species of microorganisms in the human gut, with a total weight of 1,271 grams and a total number exceeding 10 times that of human cells. The four main phyla are *Bacteroidetes*, *Firmicutes*, *Proteobacteria*, and *Actinobacteria* (11). Significant differences in gut microbiota exist across different life stages and between genders. Early life is a critical period for gut microbiota establishment, influenced by factors such as delivery method (12), breastfeeding, and antibiotic exposure (13). For instance, the colonization of *Bacteroides* and *Bifidobacterium* in infants born via cesarean section is delayed during the first few months of life compared to those born via vaginal delivery (14). Moreover, breastfeeding promotes the proliferation of *Bifidobacteria* through the secretion of specific oligosaccharides, such as 2′-fucosyllactose (15).

As individuals age, the gut microbiota becomes more complex and stable (16). Research shows that the gut microbiota in infancy gradually diversifies and becomes more stable as the child grows. Before the age of three, the alpha diversity of the gut microbiota increases annually, and the *Firmicutes/Bacteroidetes* ratio gradually rises, eventually reaching adult levels (17). As teens transition into adulthood, noticeable distinctions in gut microbiota between genders begin to surface. Post-puberty, these variations become increasingly evident, pointing to a strong link between sexual development and shifts in gut microbial composition (18). The gut microbiota exhibits dynamic shifts across life stages and genders. During puberty, pulsatile sex hormone secretion drives microbial remodeling, with testosterone supplementation inducing sex-specific changes in postpubertal mice: reduced *Firmicutes*, elevated *Bacteroidales S24_7* in females, and metabolic shifts in steroid synthesis pathways (19). These changes correlate with elevated androgen-related metabolites (e.g., cysteine-s-sulfate), suggesting microbiota-mediated modulation of sexual maturation. Crucially, prolonged testosterone exposure caused female microbiomes to adopt male-like profiles, mirroring findings that gut microbes convert glucocorticoids to androgens and that germ-free mice exhibit disrupted testosterone regulation. This supports a dual-pathway model where sex hormones regulate differentiation through both endocrine and microbiota-steroid crosstalk.

By linking life-stage-specific microbiota dynamics to gender-dimorphic metabolic traits, these observations underscore the microbiome’s role in hormone-driven developmental divergence.

### 3.2 The gut microbiota of children with CPP differs from that of normal children

Recent studies have increasingly focused on the association between gut microbiota and children with CPP. Research has shown marked disparities in the composition of gut microbiota between CPP patients and healthy individuals. A recent investigation involving 16S rRNA sequencing and untargeted metabolomics examined stool samples from 91 individuals with CPP and 59 healthy participants, revealing significant disparities in the structure and activity of their gut microbiota. In particular, the genus *Streptococcus* was significantly elevated in CPP patients, suggesting its potential as a biomarker for CPP (20). A large-scale genetic study based on Mendelian randomization analyzed genomic data from over 18,000 cases of gut microbiota and CPP, revealing significant associations between CPP and microbial groups such as *Euryarchaeota*, *Rhodospirillales*, and *Bacteroidaceae*. Notably, the protective effect of the genus *Alistipes* was especially significant, with its causality verified through sensitivity analyses (21). The results underscore the idea that the gut microbiome in children with CPP deviates markedly from that of their healthy counterparts, shedding fresh light on potential strategies for both prevention and treatment. By targeting and adjusting the gut microbiota, it may be possible to alleviate clinical symptoms and improve outcomes for children affected by CPP.

### 3.3 The gut microbiota of children with obesity-related precocious puberty differs from that of normal children

In children with obesity-related precocious puberty (OPP), the proportion of *Firmicutes* phylum is significantly increased, while the abundance of *Bacteroidetes* and *Actinobacteria* phyla is reduced (22). Among these microbiota, *Firmicutes* are typically associated with obesity and metabolic disorders, while *Bacteroidetes* are linked to a healthier metabolic state. A deep analysis of these microbiota reveals that the gut microbiota composition in children with precocious puberty tends to exhibit a higher *Firmicutes*/*Bacteroidetes* ratio, a characteristic closely related to the occurrence of obesity. This ratio may promote the onset of precocious puberty through mechanisms such as altered energy metabolism and hormone secretion.

At the genus level, beneficial microbes like *Bifidobacterium* and *Anaerostipes* decline markedly, whereas opportunistic pathogens such as *Klebsiella* rise in prevalence. Their metabolic products, such as short-chain fatty acids (SCFAs), not only participate in energy metabolism but may also promote the occurrence of precocious puberty by influencing gut barrier function, the synthesis of sex hormones, and immune system activity. Furthermore, a random forest model identified *Sellimonas* and the *Ruminococcus gnavus group* as potential biomarkers for OPP (22).

### 3.4 The gut microbiota of children with idiopathic central precocious puberty in girls differs from that of normal children

A study found that girls in the idiopathic central precocious puberty (ICPP) group had higher gut microbiota diversity and were enriched with various microbiota species associated with obesity, such as *Ruminococcus*, *Gemmiger*, *Roseburia*, and *Coprococcus*, which are linked to the production of SCFAs. Additionally, positive correlations were observed between *Bacteroides* and FSH, and between *Gemmiger* and LH (23). The increased alpha diversity of the gut microbiota and the higher abundance of SCFA-producing species in girls with precocious puberty are closely associated with elevated sex hormone levels. Specific microbiota-metabolite combinations hold potential diagnostic value (24).

## 4 Possible mechanisms of gut microbiota affecting precocious puberty

Current research suggests a possible connection between gut microbiota and the advancement of sexual maturation, particularly in cases of precocious puberty. However, the precise biological pathways through which gut microbiota influences this process remain somewhat of a mystery. That said, several plausible mechanisms could be at play, shedding light on this complex relationship. Table 1 compares gut microbiota heterogeneity across precocious puberty subtypes (CPP/OPP/ICPP), highlighting key microbial signatures, functional shifts, biomarkers and clinical implications.

**Table 1:** Comparative summary of gut microbiota alterations in different subtypes of precocious puberty.

Subtype of precocious puberty	Key microbiota changes	Microbial metabolites/functional insights	Potential biomarkers
CPP	↑ Streptococcus, ↓ Alistipes	↑ NO synthesis, ↑ estrogen reactivation via *β*-glucuronidase	Streptococcus, Alistipes
OPP	↑ Firmicutes, ↓ Bacteroidetes, ↓ Bifidobacterium, ↑ Klebsiella	Altered SCFA production, ↑ energy harvesting, ↑ LPS-induced inflammation	*Ruminococcus gnavus* group, Sellimonas
ICPP	↑ Ruminococcus, Gemmiger, Roseburia, Coprococcus	↑ SCFAs, ↑ correlations with FSH/LH levels	Gemmiger, Roseburia

### 4.1 Gut microbiome influence on sex hormone metabolism

The enterohepatic circulation of sex hormones is one of the core mechanisms through which the gut microbiota participates in precocious puberty. After hepatic metabolism, estrogens are typically excreted into the gut in an inactive glucuronide conjugate form. The “estrobolome” within the gut microbiota can hydrolyze the conjugated estrogens using *β*-glucuronidase, releasing them into their bioactive free form, allowing them to re-enter the bloodstream and activate estrogen receptors.

When gut microbiota diversity declines or there is an imbalance in microbial composition (such as a disturbance in the *Firmicutes*/*Bacteroidetes* ratio), abnormal *β*-glucuronidase activity may lead to increased levels of active estrogens in circulation. Interventions such as antibiotic overuse, high plant estrogen diets, or gastrointestinal surgeries can alter microbial structure, affecting enzyme activity and disrupting the balance of estrogen metabolism (25–27). This process may act as a potential trigger for precocious puberty by aberrantly activating the HPG axis and interfering with the physiological timing of puberty onset.

A recent study integrating microbiome and metabolomics analyses demonstrated that CPP patients exhibit distinct gut microbial dysbiosis (increased *Faecalibacterium* and decreased *Anaerotruncus*) and metabolic perturbations, including disrupted phenylalanine/tyrosine biosynthesis, TCA cycle hyperactivity, and reduced L-tryptophan levels. These alterations correlate with elevated catecholamine derivatives (e.g., homovanillic acid) and impaired serotonin synthesis, which collectively modulate GnRH secretion through neuroendocrine pathways. Furthermore, *Faecalibacterium* may enhance hypothalamic kisspeptin expression through enriched butyrate production, while the *Anaerotruncus* depletion is inversely associated with FSH levels, thereby directly linking gut microbiota-metabolite interactions to premature HPG axis activation (28).

The gut microbiota’s regulation of sex hormone metabolism also extends to the inhibition of androgen to estrogen conversion. Specific microbiota, such as *Bacteroides*, secrete 7α-dehydroxylase, which converts primary bile acids into deoxycholic acid (DCA) (29). DCA can inhibit the activity of cytochrome P450 aromatase (30), thereby blocking the conversion pathway from androstenedione to estrone, ultimately reducing the biosynthesis of estrogens (31).

The vitamin D signaling pathway and the gut microbiota form a bidirectional regulatory network that jointly influences sex hormone levels. Vitamin D deficiency leads to the suppression of vitamin D receptor (VDR) function, directly downregulating the expression of sex hormone synthases, thereby reducing the production of testosterone and estradiol. At the same time, it induces gut microbiota dysbiosis (such as reduced *Bifidobacterium* and the proliferation of pro-inflammatory bacteria), promoting the release of pro-inflammatory substances such as lipopolysaccharides (LPS) and secondary bile acids, which stimulate the premature activation of the HPG axis. Clinical studies have shown that girls with CPP often present with vitamin D deficiency, and their elevated serum free estradiol levels and accelerated uterine development may be linked to microbiota-mediated inflammatory signaling. Supplementation with vitamin D can restore gut microbiota balance (e.g., increasing the abundance of short-chain fatty acid-producing bacteria) and repair VDR signaling, thereby inhibiting the abnormal activation of sex hormones, providing a new target for the intervention of precocious puberty (32).

### 4.2 Gut microbiota influences neurotransmitter secretion

The gut microbiota can regulate the activity of the HPG axis, particularly the GnRH neurons, which are key nodes in the initiation of sexual development, by modulating the levels of neuropeptides (such as PYY and GLP-1) and neurotransmitters (such as 5-HT and GABA) in the gut. Research has revealed the precise mechanisms in which the gut microbiota controls GnRH neurons through the gut-brain axis: in patients with CPP, *Alistipes*, *Klebsiella*, and *Sutterella* species are significantly enriched in the gut. These species secrete neurotransmitters (such as serotonin and dopamine) and nitric oxide (NO), which activate the HPG axis and directly stimulate the pulsatile secretion of GnRH.

Functional prediction analysis shows a significant upregulation of the NO synthesis pathway in the gut microbiota of CPP patients (*p* < 0.001). NO not only promotes the release of gonadotropins but is also closely associated with insulin resistance and obesity, highlighting the microbiological basis for the coexistence of CPP and obesity (33). Additionally, the metabolic imbalance of estrogen sulfate in feces and glycocholate in blood further supports the regulation of the gonadal axis by the microbiota-metabolite axis (20).

### 4.3 Gut microbiota influences energy metabolism/insulin/leptin

The gut microbiota indirectly contributes to the onset of precocious puberty by regulating energy metabolism and hormone levels. Research indicates that an imbalance in gut microbiota, known as dysbiosis, can interfere with the synthesis of SCFAs, disrupt mechanisms of fat storage and energy regulation, and ultimately contribute to obesity, a known precursor to precocious puberty.

A study conducted by Li Wang et al. highlights the critical influence of gut microbiota and its SCFA byproducts in mitigating OPP in female rats, underscoring the intricate link between dysbiosis and this early developmental condition (34).

Moreover, the gut microbiota has a strong connection with the metabolism of leptin and insulin. Leptin, an important hormone secreted by adipose tissue, is responsible for regulating energy balance and appetite. Dysbiosis may lead to abnormal leptin secretion, thereby promoting premature activation of the HPG axis. These changes can also affect insulin sensitivity, induce insulin resistance, exacerbate fat accumulation, and alter sex hormone levels, thereby accelerating the onset of precocious puberty. Current research has established a clear association between obesity and early puberty in girls, with similar trends observed in boys. The mechanisms involve abnormal leptin signaling, insulin resistance, and metabolic dysregulation of the HPG axis (35).

### 4.4 Gut microbiota influences chronic inflammation

Gut microbiota dysbiosis may damage the intestinal barrier, leading to chronic low-grade inflammation, which in turn activates the HPG axis and promotes the onset of precocious puberty. Studies have shown that rats chronically exposed to blue light emitted from mobile phones, computer screens, and LED lights exhibit a significantly higher incidence of precocious puberty compared to control groups. Female rats display earlier vaginal opening and a reduced number of estrous cycles, while male rats show an earlier separation of the prepuce. Long-term exposure to blue light may disrupt gut microbiota balance, reducing beneficial bacteria and increasing pathogenic bacteria, which increases intestinal permeability. This allows more harmful substances, such as LPS, to enter the bloodstream, triggering chronic inflammation. Chronic inflammation can activate the HPG axis, resulting in elevated gonadotropin levels (LH and FSH), and promoting the onset of precocious puberty (36). For instance, lifestyle changes such as increased screen time during the COVID-19 pandemic may exacerbate inflammatory states and alter the timing of pubertal development through indirect mechanisms (37).

Furthermore, studies have shown that the LPS of Gram-negative bacteria can induce macrophage infiltration, promote the secretion of pro-inflammatory cytokines, and suppress regulatory T cells (Tregs), thereby enhancing the inflammatory response. For example, *Prevotella* species can produce TNF-*α* through the LPS mechanism, leading to the secretion of inflammatory cytokines such as IL-6 (38). In children with CPP, gut microbiota dysbiosis may enrich LPS synthesis pathways, amplifying systemic inflammation and insulin resistance. This aligns with observations in pediatric obesity, where chronic low-grade inflammation driven by adipokines (e.g., leptin, resistin) and elevated free fatty acids contributes to metabolic syndrome and endocrine disruptions, including premature HPG axis activation (39).

Therefore, maintaining gut microbiota balance and controlling chronic inflammation and oxidative stress levels may be effective strategies for preventing precocious puberty.

### 4.5 Gut microbiota influence epigenetic regulation

The gut microbiome, termed the body’s *second genome*, influences sexual development via its metabolites and microbial elements, which can influence the host’s epigenetic mechanisms and modulate gene activity. Research has shown that gut microbiota-derived metabolites, particularly SCFAs such as butyrate and propionate, inhibit histone deacetylases (HDACs), thereby enhancing histone acetylation and promoting the transcriptional activation of genes critical for metabolic and developmental processes. Additionally, microbial metabolites like choline and vitamin B12 serve as methyl donors for DNA methylation (40), stabilizing the epigenetic regulation of genes involved in neuroendocrine signaling, including those associated with GnRH secretion.

A Mendelian randomization study has, for the first time, provided genetic evidence suggesting that increased abundance of Alistipes may be associated with a reduced risk of CPP by up to 80%, indicating a potential protective role in pubertal regulation. Potentially through DNA methylation-mediated silencing of GnRH promoter regions. This protective effect aligns with findings that gut microbiota composition modulates the gut-brain axis, influencing HPG axis activity via epigenetic reprogramming of neuroendocrine pathways (41, 42).

For instance, SCFAs produced by *Akkermansia muciniphila* enhance intestinal barrier integrity and reduce systemic inflammation, indirectly suppressing oxidative stress and aberrant DNA methylation linked to premature activation of puberty-related gene (42). These mechanisms highlight the bidirectional interplay between microbial metabolites, epigenetic modifications, and sexual maturation, offering novel insights into microbiota-targeted interventions for pubertal disorders.

**Figure 1:**
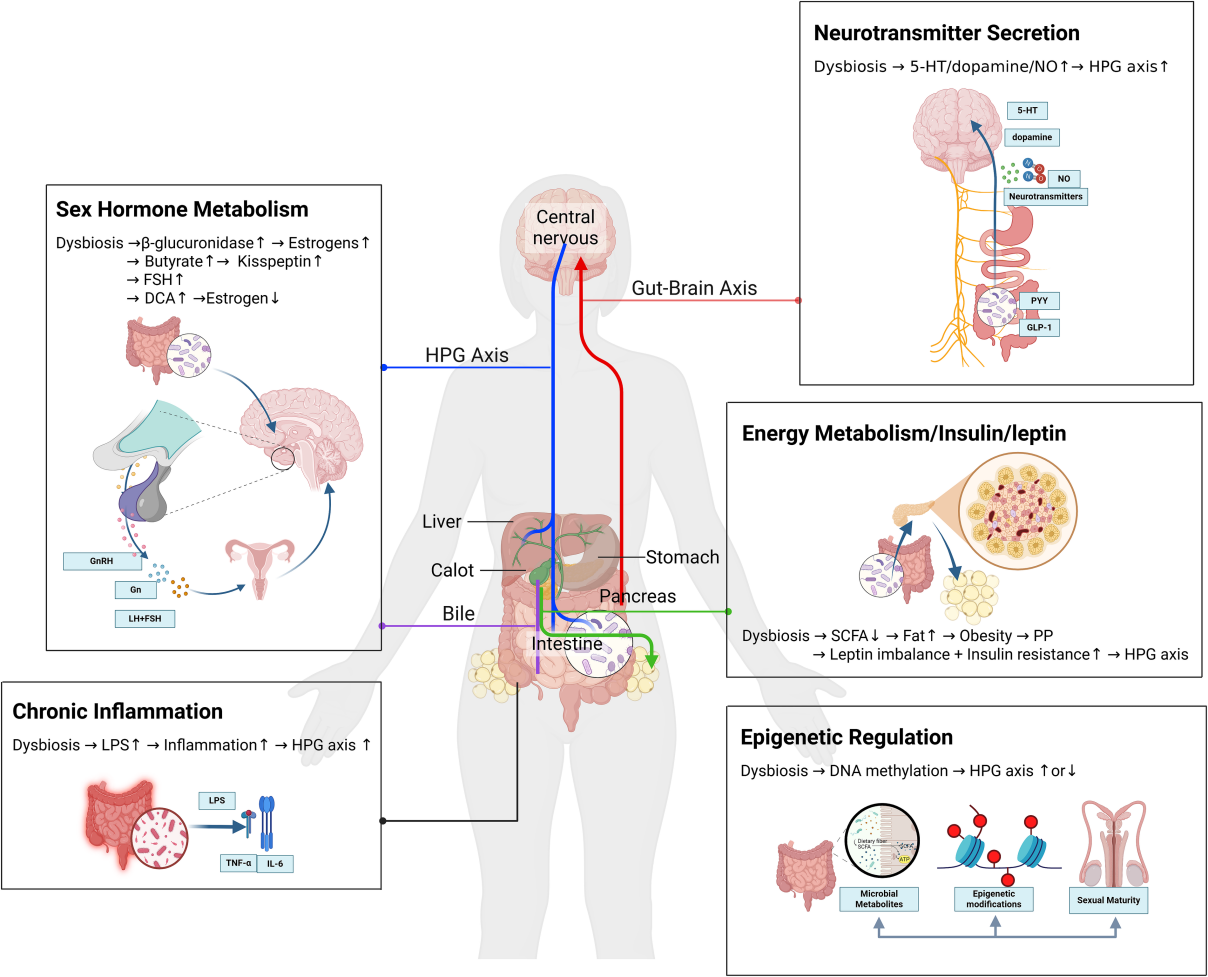
Gut microbiota dysbiosis in precocious puberty. A mechanistic network of metabolic, neuroendocrine, and epigenetic crosstalk.

To visually summarize these pathways, we have constructed a mechanistic diagram (Figure 1) that integrates the key interactions between gut microbiota dysbiosis, metabolic perturbations, neuroendocrine signaling, and epigenetic modifications in the pathogenesis of precocious puberty.

## 5 Prospects for the clinical application of regulating gut microbiota in the treatment of children with precocious puberty

### 5.1 Probiotic therapy

Probiotics have broad application prospects in regulating the gut microbiota. Through fecal microbiota transplantation (FMT) and animal model studies, the key role of gut microbiota in OPP has been confirmed, providing a theoretical basis for probiotic therapy. Soy isoflavones (SI) and their metabolite daidzein (DAI), as phytoestrogens, significantly alter gut microbiota composition (e.g., increased *Christensenella* and *Enterococcus* abundance) and reduce SCFAs like butyrate, isovalerate, and hexanoate in female mice, thereby accelerating pubertal onset via HPG axis activation (37). Probiotic treatment reverses DAI-induced dysbiosis, restores SCFAs (particularly butyrate) to baseline levels, and delays vaginal opening timing and uterine weight, indicating its anti-precocious puberty effects through reshaping microbial metabolic functions (43).

Furthermore, early-life stress impacts pubertal timing via sex-dependent mechanisms: maternal separation stress causes earlier puberty in females but delays it in males. Probiotics (containing *Lactobacillus* and *Bifidobacterium*) normalize pubertal timing in both sexes, potentially by modulating HPA/HPG axis crosstalk and microbiota-dependent hormone metabolism (44).

Notably, a 2024 clinical cohort study demonstrated that girls with OPP exhibited a marked reduction in *Bifidobacterium* abundance compared to healthy controls. Probiotic supplementation targeting *Bifidobacterium* not only restored its colonization but also ameliorated estrogen imbalance and suppressed LH levels, suggesting microbiota-mediated endocrine regulation as a therapeutic pathway (45). A random forest model further identified *Sellimonas* and *Anaerostipes* as discriminatory taxa for OPP diagnosis, highlighting their potential as non-invasive biomarkers for precision probiotic interventions (22).

Although these preclinical and clinical findings underscore the translational potential of probiotics, current evidence remains limited by small sample sizes and heterogeneous intervention protocols. Large-scale, multi-center randomized trials are imperative to validate long-term efficacy, optimize strain-specific formulations, and assess safety profiles in diverse pediatric populations.

### 5.2 Fecal microbiota transplant

FMT, as an emerging intervention strategy, has shown preliminary effectiveness in the treatment of various diseases. A study demonstrated that FMT from high-fat diet (HFD) mice to normal diet mice led to precocious puberty in the recipient mice. Microbiota analysis revealed enrichment of pro-inflammatory genera such as *Streptococcus* and *Bacillus* in the recipient mice, suggesting that the microbiota may activate the gonadal axis through inflammatory signaling or metabolic products (such as DCA), thus promoting the development of precocious puberty (46).

In another animal experiment, FMT from female rats treated with glycyrrhizin significantly delayed vaginal opening and reduced GnRH expression in germ-free mice (47), providing new experimental evidence for microbiota-targeted interventions. There is also a study that indirectly proves this view: maternal high-fat diet (MHFD) during lactation altered offspring’s gut microbiota, causing obesity, insulin resistance, and earlier vaginal opening (3–4 days). Co-housing MHFD offspring with normal-diet offspring (via coprophagia) reversed microbiota dysbiosis, restoring puberty timing and insulin sensitivity (48).

These studies demonstrated that gut microbiota reconstitution improves precocious puberty independently of obesity, providing critical preclinical evidence for FMT in clinical translation. Although FMT in humans is still in the exploratory phase, these studies lay the experimental foundation for its clinical translation, with further validation and standardization required in the future.

### 5.3 The diet-microbiota co-intervention strategy

Dietary influences on gut microbiota are well-established. Beyond inducing compositional and functional alterations in gut microbiota itself, microbial metabolism of dietary phytoestrogens may play a pivotal role in precocious puberty pathogenesis. For instance, daidzein from soy products undergoes microbial fermentation and metabolism to generate metabolites with differential estrogenic activities. Notably, *Escherichia coli* has been demonstrated to convert 8-OHD into 6-OHD, a metabolite exhibiting potentially stronger estrogenic activity (49), providing novel insights into how dietary components may promote sexual precocity through microbial modulation.

Animal studies further elucidate the mechanism whereby HFD induces precocious puberty via gut dysbiosis. Post-weaning mice subjected to persistent HFD exhibited significant enrichment of (e.g., *Lachnoclostridium* and *Desulfovibrio*) accompanied by elevated serum estradiol, leptin, and hypothalamic GnRH levels, culminating in significantly advanced vaginal opening (46). Furthermore, MHFD during gestation or soy isoflavone-containing formula feeding may predispose offspring to obesity and precocious puberty through microbial alterations, whereas ≥ 6-month breastfeeding helps maintain microbial homeostasis and reduce obesity risk, thereby delaying pubertal onset (35, 50). Notably, reduced carbohydrate metabolic pathways in the gut microbiota of children with precocious puberty have been reported (51).

Consequently, low-sugar/high-fiber diets not only effectively modulate microbiota composition and mitigate obesity-related dysbiosis, but also delay puberty initiation through improving glycemic control and insulin sensitivity. Collectively, integrated dietary-microbial intervention strategies present novel therapeutic avenues for precocious puberty prevention and management.

### 5.4 Microbiota-targeted therapeutic development

The development of microbiota-targeted pharmaceuticals has emerged as a focal area in gut microbiome research. These therapeutic agents modulate gut microbial composition and functionality to suppress pathobiont overgrowth and restore microbial ecological balance, thereby regulating hormonal profiles and immune responses to mitigate precocious puberty progression. Employing high-throughput screening platforms and animal models, researchers have preliminarily identified puberty-associated probiotic strains, including specific *Lactobacillus* and *Bifidobacterium* species, which exert their effects through dual mechanisms of intestinal barrier enhancement and immune system modulation on gonadotropin secretion.

Notably, the natural polyphenol epigallocatechin gallate (EGCG) has demonstrated efficacy as a microbiota-directed intervention. It regulates *Akkermansia muciniphila* abundance, suppresses aberrant tryptophan metabolism, and downregulates hypothalamic *Cyp1b1* expression, effectively delaying HFD-induced precocious puberty (52). This exemplifies the potential of phytogenic microbiota modulators. With accumulating clinical evidence, this field is poised to advance from microbial strain selection to phase I clinical trial implementation, bridging translational gaps in puberty-related microbiome therapeutics.

## 6 Conclusion and future perspectives

In recent years, the role of the gut microbiota in the onset of precocious puberty has become an emerging research focus. The connection of gut microbiota with precocious puberty provides fresh perspectives and possible intervention approaches. The microbiota participates in the development of precocious puberty through various mechanisms. Based on these mechanisms, probiotic therapy, FMT, diet-microbiota co-intervention strategy, and microbiota-targeted drugs represent promising directions for the prevention and treatment of precocious puberty. Future research should further explore the specific mechanisms linking the gut microbiota with precocious puberty, and develop more effective intervention strategies to reduce the incidence of precocious puberty and improve the health outcomes of affected children.

Despite growing interest in the gut microbiota’s role in precocious puberty, several research gaps remain. Most current studies are observational, making it difficult to determine causality between microbial changes and precocious puberty onset. Additionally, heterogeneity in diagnostic criteria, sequencing methods, and population characteristics limits comparability across studies. Functional mechanisms, especially those involving microbial metabolites and host interactions, are still underexplored. Future research should prioritize longitudinal and interventional studies with standardized protocols to clarify causal links and inform targeted therapies. Individual variability in gut microbiota composition can influence therapeutic efficacy and complicate standardization. Moreover, safety and regulatory concerns surrounding FMT—such as the risk of pathogen transmission, long-term effects, and ethical considerations—must be carefully addressed. Robust clinical trials, rigorous donor screening, and standardized treatment protocols are essential to ensure both safety and effectiveness in pediatric populations.
